# Nanosystems for Improved Targeted Therapies in Melanoma

**DOI:** 10.3390/jcm9020318

**Published:** 2020-01-23

**Authors:** Cristina Beiu, Calin Giurcaneanu, Alexandru Mihai Grumezescu, Alina Maria Holban, Liliana Gabriela Popa, Mara Mădălina Mihai

**Affiliations:** 1 Department of Oncologic Dermatology-“Elias” Emergency University Hospital, “Carol Davila” University of Medicine and Pharmacy, 020021 Bucharest, Romania; cristina.popescu@drd.umfcd.ro (C.B.); calin.giurcaneanu@gmail.com (C.G.); lilidiaconu@yahoo.com (L.G.P.);; 2 Department of Science and Engineering of Oxide Materials and Nanomaterials, Faculty of Applied Chemistry and Materials Science, University Politehnica of Bucharest, 1–7 Polizu Street, 011061 Bucharest, Romania; grumezescu@yahoo.com; 3 Department of Microbiology and Immunology, Faculty of Biology, University of Bucharest, 030018 Bucharest, Romania

**Keywords:** melanoma, targeted therapy, nanotechnology, immunotherapy, nanoparticles

## Abstract

Melanoma is one of the most aggressive forms of skin cancer, with limited therapeutic options. Since its incidence has been rapidly rising in recent years, the study of new targeted therapeutic strategies has increased. The implication of nanoscience in the development of alternative targeted therapies for melanoma has multiple benefits and could significantly improve the outcome of melanoma patients. In this paper, we review the most recent progress in the field of targeted therapies, emphasizing the impact of nanoscale materials on the targeting and controlled release of anti-tumor drugs. The applications of nanomedicine in the management of melanoma are extensive and refer to sentinel lymph node mapping, chemotherapy, and RNA interference; each of these applications harboring the potential to develop efficient and personalized diagnostic techniques and therapies. Further research, especially in clinical trials, is needed to establish whether fighting melanoma on the nanoscale level represents the key to reaching a critical inflection point in mankind’s battle with metastatic melanoma.

## 1. Introduction

The incidence of melanoma, the most aggressive form of skin cancer, is alarmingly rising on a global scale. The American Cancer Society suggests a worrying estimate for 2019 in the U.S. of 96,480 newly diagnosed cases of invasive melanoma and 7230 deaths from advanced melanoma [1].

The treatment of melanoma has evolved significantly over time, from surgery to chemotherapy, to radiation therapy, and nowadays to modern targeted therapy. When melanoma is diagnosed at an early stage, surgical excision may be curative. However, in the setting of advanced melanoma complex surgical and medical treatment is necessary and the clinician is often faced with difficult therapeutic challenges, long-term responses being difficult to achieve. However, recent advances in the modulation of immune responses against cancer have changed paradigms, and with the first regulatory approval of a novel immunotherapeutic agent targeting an immune checkpoint, cytotoxic T-lymphocyte-associated protein 4 (CTLA-4), ipilimumab, the future of cancer care was changed forever. This very moment has subsequently led to the approval of over ten different immunotherapeutic agents, including other checkpoint inhibitors.

The main available medical therapies of melanoma are:

- checkpoint inhibition immunotherapies: programmed cell death 1 (PD-1) or programmed cell death ligand 1 (PD-L1) inhibitors (nivolumab, pembrolizumab) alone or in combination with anti-cytotoxic T-lymphocyte-associated protein 4 (CTLA-4) antibodies (ipilimumab);
- molecularly targeted therapies used in patients with driver mutations, against the mitogen-activated protein kinase (MAPK) pathway, with BRAF (v-raf murine sarcoma viral oncogene homolog B1) and MEK (mitogen-activated protein kinase) inhibitors (vemurafenib, dabrafenib, encorafenib, and trametinib, cobimetinib, binimetinib, respectively) in patients harboring a V600 mutation in the BRAF gene, and tyrosine-protein kinase (KIT) inhibitors (imatinib);
- tumor vaccines;
- gene therapy, such as locoregional immunotherapy with oncolytic viruses (talimogene laherparepvec- T-VEC);
- T cell directed therapies;
- other non-specific approaches (radiotherapy, cytotoxic chemotherapy, photothermal therapy, photodynamic therapy, electrochemotherapy, and others) indicated only in a few subsets of patients due to their questionable benefits for the patient’s outcome [2].

Despite the continuous research and development of novel antineoplastic therapies, melanoma remains a disease that continues to outpace the solutions. The efficacy of the currently available melanoma therapies is limited by multiple drawbacks. The drugs’ interaction with biological fluids and membranes, as well as the heterogeneous tumor immunology and vasculature, are possible explanations for drug resistance [3,4].

Leading scientists focus their research on developing innovative nano-technologies that have the potential to increase therapeutic accumulation in targeted malignant melanocytes and enhance drug efficiency, while lowering systemic toxicity. The properties of nanomaterials (their shape, size, and interface) can be optimized in order to overcome all these barriers and bridge this gap. This has led to the development of ultra-small drug delivery platforms driven by nano-technology.

Most of the research on the subject is currently preclinical. Officially, on the clinicaltrials.gov database there are 2415 clinical trials on melanoma worldwide, but only ten investigating the use of nanotechnology in this type of cancer.

In this article, we aim to review the intensive research carried out at the interface between nanotechnology, medicine, biology, and chemistry, in the effort to enable nanosystem-based treatments for melanoma patients.

## 2. Nanotechnology: Mechanisms and Available Nanoparticles in Melanoma Therapy

### 2.1. Mechanisms of Drug Targeting through Nanosystems

Nanostructured materials have been comprehensively studied, with the purpose of enabling precision targeted drug delivery for melanoma treatment. They have the ability to navigate the body’s maze and to deliver drugs directly into a diseased cell only, without affecting healthy cells. This concept of drug targeting through nanosystems results in higher efficacy and fewer side effects. The results can be achieved through both passive and active targeting [5,6].

#### 2.1.1. Passive Targeting

**Figure 1:**
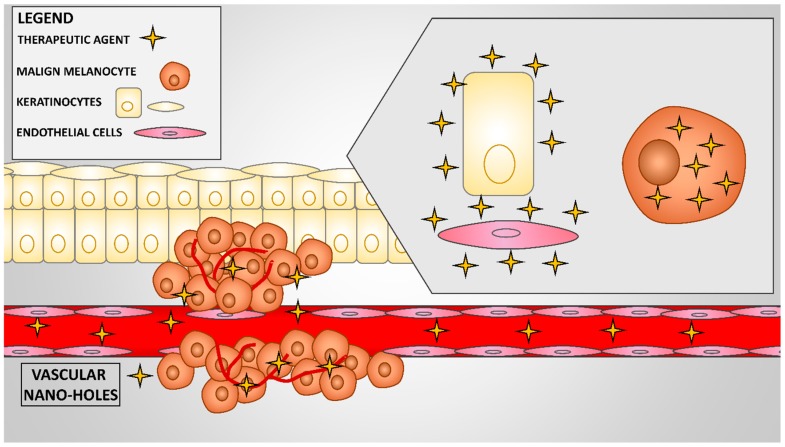
Passive targeting of nanotechnology-based drug delivery systems in melanoma. The figure shows the enhanced permeability (through nano-holes) and retention of nanoparticles in malignant tissues. The nanoparticles have no/little effect on healthy cells (keratinocytes, endothelial cells, and others).

The EPR (enhanced permeability and retention) effect is a characteristic feature of tumor neovascularization and it represents the basis of nanotechnology-based drug delivery systems (Figure 1). The tumor blood vessels differ very much from the ones in healthy tissues [3]. In cancer sites, blood vessels are discontinuous, resembling pipes with nano-holes. Therefore, nanoparticles can exit the circulation and penetrate the tumor interstitial space [3]. Furthermore, lymphatic filtration is poor within tumors, which facilitates drug accumulation [3]. Thus, the high permeability and increased retention processes lead to the accumulation of nanoparticles in solid tumors and to the release of therapeutic agents into malignant cells [3,7,8]. Contrarily, in healthy tissues, the blood vessels do not possess the same large fenestrations and the nanoparticles cannot exit the blood circulation and enter healthy tissues [3].

These findings gave birth to an emerging field investigating different types of nanotechnologies capable of actually accumulating a medicine in a diseased area rather than affecting the healthy areas of the body.

#### 2.1.2. Active Targeting

Active targeting (also called ligand-based targeting) represents a similar strategy but with increased specificity in binding to certain tissues. This is due to the attachment of targeting ligands (such as antibodies) to the nano-carriers. Ligands are able to recognize surface receptors on the specific targeted cells and effectively bind to the receptor site, increasing the drug’s specificity.

### 2.2. Available Nanoparticles in Melanoma Therapy

The most investigated nanoparticles effective in melanoma therapy are: polymeric nanoparticles, liposomes, inorganic nanoparticles, carbon nanotubes, and dendrimers.

#### 2.2.1. Polymeric Nanoparticles

Polymeric nanoparticles are being researched extensively as drug delivery vehicles due to a number of factors, which include: (1) flexibility; (2) controlled release properties; (3) good biocompatibility and biodegradability; and (4) their ability to be used as multifunctional carriers [9].

Various types of polymeric nanoparticles can be synthesized depending on the polymer’s properties and its applications, namely nanospheres and nanocapsules, polymeric micelles, polymersomes, dendrimer-based micelles, and polymer–drug conjugates [10].

#### 2.2.2. Liposomes

Liposomes are specialized lipid vesicles that possess a central aqueous compartment surrounded by a bilayer hydrophobic structure of phospholipids, similar to cell membranes [5]. Nanostructured liposomal systems offer an excellent opportunity as drug vehicles, since they can carry both hydrophilic drugs (through encapsulation in their central compartment) and lipid-soluble drugs (embedded in the phospholipids tail) [5].

They are able to increase drug circulation time through various mechanisms, such as size adjustment (under 100 nm) or functionalization (e.g., PEGylation) [5]. In this way, detection by the immune system is avoided and an extended drug release is achieved [5]. Liposomes also possess excellent internalization and diffusion properties, due to their ability to fuse with cell membranes [5]. Additionally, their reactive external lipid layer can be conjugated with ligands for more specific tissue targeting [11].

#### 2.2.3. Inorganic nanoparticles

Noble metals such as gold, silver, and platinum biocompatible nanomaterials have applications in melanoma diagnosis and treatment.

Gold nanoparticles (AuNPs) present with major biomedical advantages, such as: synthetic accessibility, versatility in size, shape or surface chemistry, valuable biocompatibility and stability, surface plasmon resonance [12].

Due to the above properties, AuNPs have been extensively investigated for various functions, such as: (1) delivery vehicles for drugs and small interfering RNA (siRNA); (2) photodynamic and photothermal therapeutic utility.

AuNPs are also suitable for an active delivery model, as they can be conjugated with antibodies against cancer markers. In an experiment on human melanoma cells, Choi B.B.R. et al. (2017) conjugated AuNPs with phosphorylated focal adhesion kinase (p-FAK), a protein highly expressed in melanoma cells, and combined the treatment with cold plasma treatment [13]. G361 human melanoma cells were selectively destroyed, with no harm to the healthy cells [13]. However, further research is necessary to explore the interaction between AuNPs and plasma [13].

Lomelí-Marroquín D. et al. (2019) achieved the synthesis of bimetallic silver/gold nanoparticles, with the advantages of an increased biocompatibility, usually associated with AuNPs, and of a decreased toxicity of silver nanoparticles (AgNPs) [14]. The product exhibited a dose-dependent anticancer effect on melanoma cells in vitro [14].

Platinum nanoparticles (PtNPs) have also been used in preclinical trials. They showed good biocompatibility, as well as anti-inflammatory effects due to their enzymatic properties against reactive oxygen species [15,16].

#### 2.2.4. Carbon Nanotubes

Carbon nanotubes (CNTs) are molecular tubes formed from one or more rolled-up sheets of graphene (an individual layer of carbon atoms). They are classified as single-walled (SWNT) and multi-walled nanotubes (MWNT) [17]. CNTs are not soluble in aqueous media, so the process of functionalization (modification of the surface of CNTs through attaching other chemical groups to the tip or sidewall) is essential for their biological and biomedical applications [17].

#### 2.2.5. Dendrimers

Dendrimers are three-dimensional synthetic polymers with a branching tree-like structure [18]. The word “dendrimer” is derived from the Greek “dendros”, meaning “tree”, and “meros”, meaning “part of” [19].

Structurally, dendrimers are constructed by the stepwise connection of a central core, multiple branched arms, and peripheral groups. They can be synthesized through two approaches: (i) a convergent approach (branches are synthesized separately and subsequently linked to a central core) or (ii) the divergent approach (the branches grow directly from the central core) [18].

Their main characteristics (such as size, shape, surface chemistry, solubility, architecture, and chemical composition) can be easily optimized in the engineering process [18]. Thus, due to their topology, researchers can achieve highly precise control over the dendrimer structure at the molecular level [18]. Their unique structure also allows the possibility of introducing multiple functionalities in various locations of only one dendrimer (e.g., on the surface, on the core, at the branching points, and even encapsulated between the branches), making them perfectly suitable for both drug entrapments in the central structures, as well as surface linking for targeted delivery [18].

## 3. Nanosystems for Improved Targeted Therapies in Melanoma

In recent years nanotechnology has developed considerably, with extensive biomedical applications, also allowing the engineering of better therapies in melanoma. Multiple in vivo and in vitro studies showed significant improvements of almost each standard melanoma therapy by the association of nanoparticle delivery systems. In this subchapter we aim to review the multiple applications of nanotechnology in the treatment of melanoma, starting from the advantages and drawbacks of available therapies towards their improvement by the use of nanosystems.

### 3.1. Melanoma Immunotherapy

Cancer immunotherapy (passive or active) can specifically and persistently enhance the immune response to fight tumor cells. Over the years, promising results have been achieved in preclinical and clinical studies with several types of immunotherapeutic agents: monoclonal antibodies, immune checkpoint inhibitors, non-specific immunomodulatory agents, whole tumor cancer vaccines, and others [20,21].

#### 3.1.1. Immune Checkpoint Inhibitors

Immunological drugs have changed the therapeutic landscape of advanced melanoma.

The most significant advantages of checkpoint inhibition immunotherapy are the long-term response, the prolongation of overall survival, and progression-free survival.

Dual therapy with nivolumab plus ipilimumab seems to achieve higher response rates and prolong survival more than nivolumab monotherapy in metastatic melanoma patients, regardless of the BRAF status [22,23]. According to Larkin J. et al. (2019), a sustained long-term overall survival at five years was obtained with immunotherapeutic agents in the following descending order: nivolumab plus ipilimumab (more than 60.0 months), nivolumab alone (36.9 months), ipilimumab alone (19.9 months) [23]. In the KEYNOTE-006 trial, pembrolizumab showed superiority over ipilimumab after almost five years of follow-up with respect to the median overall survival (32.7 months versus 15.9 months) and the median progression-free survival (8.4 months versus 3.4 months) [24].

Nivolumab is indicated as an adjuvant treatment in all patients diagnosed with stage IIIA-IIID melanoma or totally resected stage IV melanoma. Nivolumab and ipilimumab combined treatment also shows promising results as neoadjuvant therapy in patients with macroscopic stage III melanoma [25].

Despite these encouraging results, the recent CheckMate 067 study showed that 52% of patients reached a five-year overall survival with immunotherapy combination strategy [23]. One in two patients with metastatic melanoma still require alternative therapies [23]. Several mechanisms involved in the development of resistance to immunotherapy have been described, among them the low tumoral immunogenicity, limited T cell immune response, and others [26].

Another major aspect to be considered is that responses to immunotherapy typically develop slowly and patients may experience transitory exacerbations of disease before the objective therapeutic response is achieved.

Moreover, patients undergoing immunotherapy often experience an array of immune-related adverse events (irAEs) that range from mild to serious, or even potentially fatal reactions. The most frequently encountered irAEs are dermatological (vitiligo-like leukoderma, lichenoid dermatitis, psoriasiform dermatitis, or even potentially life-threatening conditions such as Stevens Johnson Syndrome), diarrhea/colitis, hepatitis, pneumonitis, and endocrine toxicities (requiring hormonal supplementation). In the KEYNOTE-006 trial, patients treated with pembrolizumab, severe grade 3–4 treatment-related adverse events occurred in 96 (17%) of 555 patients in the combined pembrolizumab groups and in 50 (20%) of 256 patients in the ipilimumab group [24].

Moreover, with more effective combined scheme therapy (nivolumab and ipilimumab), the incidence and severity of adverse effects is further increased. Mason R. et al. (2019) reported that 67% of patients developed treatment-related adverse events and 38% of individuals suffered from severe, grade 3–5 reactions [27].

The treatment of moderate–severe irAEs requires discontinuation of the checkpoint inhibitor and the administration of intravenous corticosteroids (1–2 mg/kg dose). The treatment can be resumed afterwards, but data on the results of this approach are limited [28].

Benson Z. et al. (2017) proposed the concept that tumor response to immunotherapy may be increased by the pre-exposure of malignant tissues to nanotechnology or other compounds able to trigger immunogenicity, to achieve epigenetic modulation or inhibit cell cycle progression [29]. Intensive research is carried out on gene therapy with nano-complexes of tumor associated antigens-encoding RNA, with the aim of increasing the immunogenicity of melanoma, as it will be described below. However, there is scarce information on the combination of gene therapy with immune checkpoint inhibitors. Probably this will represent the focus of research in the years to come.

Another application of nanotechnology in immunotherapy was highlighted by Iwamoto N. et al. (2016), who developed a method of pharmacokinetic study using nano-surface and molecular-orientation limited (nSMOL) proteolysis to quantify nivolumab in human plasma, probably useful for the quantification of other checkpoint inhibitor agents [30].

#### 3.1.2. High-Dose Interleukin-2 (IL-2)

IL-2 was the first immunotherapy to influence the outcome of advanced melanoma patients, but its harsh toxicity with cardiovascular, respiratory, and infectious complications restrained its use. It is now considered an option in only a few selected patients [2]. The potentially lethal immunotoxicity is caused by the stimulation of circulating leukocytes and the “cytokine storm” following it [31].

A very interesting ongoing phase 1/2 clinical trial is investigating the efficiency of an IL-2 pegylated molecule (Bempegaldesleukin) in combination with nivolumab for the treatment of melanoma, with promising primary results [32].

Wu T. et al. (2019) ingeniously developed a combinatorial drug delivery system by absorbing interleukin-2 (IL-2) in doxorubicin (DOX)-loaded vesicles [33]. The nano-complex suppressed tumor growth in melanoma mice with negligible systemic toxicity [33].

In order to reduce the severity of adverse effects, Xie Y.Q. et al. (2019) developed a complex delivery system, binding redox-responsive IL-2/Fc nanogels to the surface of adoptively transferred T cells [34]. Interestingly, the cytokine nanogels are able to selectively release IL-2 in response to the activation of T cell receptors after antigen recognition in tumors [34]. In a melanoma mouse model the delivery system showed improved efficiency, without obvious toxicity [34].

Immunoliposome delivery systems of IL-2/anti-CD137 can achieve equivalent anti-tumor activity compared to the free molecular agents, with low or even absent systemic toxicity [31]. Zhang Y. et al. (2018) showed that the attachment of IL-2 and anti-CD137 on the surface of liposomes can quickly accumulate in melanoma tumors and, therefore, minimize the systemic exposure and lower the risk of toxicity [31].

#### 3.1.3. Tumor Vaccines

Cancer immunotherapy can specifically and persistently enhance the immune response. A series of preclinical and clinical studies have explored the benefits of whole tumor cancer vaccines [20,21]. They can induce immunity against a wide variety of antigens [21] and can be obtained more easily compared to the selection of specific peptides [21]. The use of neoantigen vaccines for melanoma will probably be expanded in the future [35].

Although they are currently under intensive investigation, most cancer vaccines have low to moderate clinical effects and are unsuccessful in eliciting relevant results [36], probably due to the important knowledge gaps regarding of the mechanisms underlying oncogenesis.

A current point of interest is the design of personalized micro- and polymeric nanoparticle cancer vaccines, to co-deliver melanoma antigens, as well as therapeutic adjuvants.

Tumor membrane-coated nanoparticles share some similarities with whole tumor vaccines, since they induce immune responses against multiple tumor antigens, without the need to select specific peptides [21].

Fontana F. et al. (2019) designed a cancer nanovaccine based on biohybrid (TOPSi@AcDEX) nanoparticles coated with membranes derived from tumors cells that promote antigen cross-presentation, enabling the maturation of immature monocyte-derived dendritic cells [21]. The nanoparticles are able to degrade rapidly in physiological fluids with the release of silicic acid [37] and increase the expression of major histocompatibility complexes [38].

The activated antigen-presenting cells contribute to the stimulation of Th1 lymphocytes, followed by CD8 T cells activation, and the secretion of interferon-gamma (IFN-γ) [21]. Moreover, nanovaccines promote the presentation of antigens to memory T cells through the cross-dressing of membranes [39,40]. Nanovaccines can also be combined with cytokines to enhance immune cell activation [41].

Liu W.L. et al. (2019) proposed a cytomembrane nanovaccine derived from dendritic cells and cancer cells, allowing the nanoparticles to exert an effect similar to the physiological antigenic presentation and lead to an efficient T cell activation [36].

Conniot J. et al. (2019) from Tel Aviv University showed that nanoparticles could potentially be used in the treatment of melanoma, similar to the use of vaccines for viral diseases [42]. The researchers ingeniously combined passive and active immunotherapy. The nanoparticles, made of biodegradable polymer and sized at 160–190 nanometers, were individually packed with two peptides expressed in melanoma cells: Melan-A/MART-1(26-35(A27L)) major histocompatibility complex class I (MHCI)-restricted peptide (MHCI-ag) and the Melan-A/MART-1(51-73) MHCII-restricted peptide (MHCII-ag), directed towards the MHC class I and class II antigen presentation pathways [42]. They grafted the nanoparticles with mannose receptors, making them capable of ligand-mediated (active) targeting of dendritic cells [42]. The mannosylated nanovaccines were combined with an anti-PD-1 antibody (αPD-1) for immunosuppression blockade and an anti-OX40 antibody (αOX40) for T cell stimulation [42]. After injecting the nanovaccines into mice models, they examined their effectiveness and found that it had both a prophylactic effect, preventing the development of melanoma in healthy immunized mice, and a therapeutic effect, significantly delaying the progression of the disease even in late-stage melanoma mice [42].

Further research is mandatory to establish whether it is safe to power-up melanoma approach with nanovaccines in humans.

### 3.2. Molecularly Targeted Therapy

Nowadays, combined BRAF and MEK inhibition therapy has replaced BRAF inhibition alone. The available combinations include dabrafenib plus trametinib, vemurafenib plus cobimetinib, and encorafenib plus binimetinib [43,44].

The major advantage of such combined treatments is the capacity to induce an accelerated tumor regression in most of the patients with BRAF V600 mutation-positive melanoma. This is why they are now recommended as first line therapy, especially in patients in whom a quick objective response to therapy is crucial, such as symptomatic patients with high tumor load and poor prognosis factors (e.g., high lactate dehydrogenase seric level). Patients who do not fit these criteria of severity should benefit from immunotherapy as first line treatment, regardless of the BRAF status [43].

Moreover, when compared to the administration of single-agent BRAF inhibitor therapy, response rates and survival periods are improved, while resistance and toxicity are lowered [45].

Acquired resistance mechanisms still represent the major drawback, since they are only delayed by the combined therapy [45]. Studies also highlighted an array of important side effects that may strongly influence the patient’s outcome after the systemic administration of BRAF/MEK inhibitors, such as pyrexia and chills, reduced cardiac ejection fraction, and increased alanine aminotransferase level in dabrafenib/trametinib-treated patients [43]; bleeding disorders, visual impairment, increased creatine kinase seric level, and dermatologically adverse effects (severe phototoxicity, palmo-plantar kerato-derma) in patients undergoing treatment with vemurafenib/cobimetinib [44].

Another very important problem is the unclear sequencing of treatments in BRAF-positive patients. A current major controversy is related to the initial decision between immunotherapy and molecularly targeted therapy, as well as the benefit of switching to immunotherapy after BRAF/MEK inhibitors as first line therapy. Recent data showed that the action on symptom-free melanoma brain metastases of different immunotherapies is lower in patients previously treated with BRAF/MEK inhibitors [46]. Moreover, Mason R. et al. (2019) recently reported a significant difference in therapeutic response to immunotherapy (nivolumab plus ipilimumab) in naïve patients compared to BRAF/MEK failure patients: an overall objective response rate of 57% and 21%, respectively, and a median progression-free survival of 11.0 months and 2.0 months, respectively [27].

Starting from the rationale that BRAF and MEK inhibitors have the ability to increase T cell responses through a variety of immunological changes, researchers combined BRAF/MEK inhibitors and anti-PD1 antibodies to achieve a synergistic effect in the treatment of metastatic melanoma. The study is named COMBI-I and preliminary data have shown that more than 40% of patients had a confirmed complete response, while a large proportion, 78% of patients, expressed adverse events, grade 3 or higher [47].

Ramesh A. et al. (2019) went even further and studied an improved nano-model of combined therapy [48]. They engineered a delivery nanosystem of PI3K- and MAPK-inhibitors associated with anti-PD-L1 immune checkpoint immunotherapy and observed a synergistic effect, with better anti-melanoma efficacy and minimal T cell cytotoxicity [48].

The same study group showed that nanotechnology can also be used to modulate tumor microenvironment together with kinase inhibition [49]. Supramolecular nanoparticles were used to inhibit colony-stimulating factor 1 receptor (CSF1R) and MAPK signaling and to further block the pro-tumorigenic phenotypes of tumor-associated macrophages [49]. They also detected a significant macrophage repolarization towards an anti-tumorigenic phenotype [49].

### 3.3. Cytotoxic Chemotherapy

The use of cytotoxic chemotherapy in the treatment of advanced melanoma is extremely limited.

Melanoma is known to be chemotherapy-resistant, with a median duration of response of approximately four to six months [50]. In consequence, chemotherapeutic agents (e.g., dacarbazine and its prodrug temozolomide, carboplatin/paclitaxel, fotemustine) are rarely used in the treatment of advanced melanoma, mainly in patients in whom all other therapeutic options have been exhausted [2]. Chemotherapy’s failure has been largely attributed to its non-specific distribution and lack of accumulation in the tumor. However, nanosized chemotherapeutic carriers are showing promising results. In a phase III trial, Nab-paclitaxel, an albumin-bound paclitaxel, showed significantly increased progression-free survival in metastatic melanoma patients when compared to dacarbazine alone [51].

Functionalized CNTs have been proven to boost the effectiveness of chemotherapy in melanoma cells. Chaudhuri P. et al. (2010) showed that a SWNT conjugated with doxorubicin (DOX) reduced the side-effects of chemotherapy drugs while increasing their bioavailability, thus generating better drug efficacy in a B16-F10 laboratory melanoma model [52].

CNTs were also used for transdermal delivery of DOX. Back in 2010, scientists used multi-walled and double-walled functionalized nanotubes (through PEGylation—with Polyethylene glycol) for the transdermal delivery of indomethacin (hydrophilic, basic drug) and doxorubicin (hydrophobic, acidic drug) [53]. Results showed that CNTs represent promising material as a drug bio-delivery system [53].

In a recent study on melanoma mice models, doxorubicin-loaded liposomes were grafted with a T cell receptor (TCR)-like antibody targeted to melanoma antigen A1 (MAGE-A1) [11]. These immune-liposomes showed enhanced pharmacokinetics in vivo and induced an important antitumor response in vitro [11].

The efficacy of liposomes in anti-cancer therapies can also be increased by using pH as a trigger for drug release, considering the 6.5 pH of tumor tissues. In melanoma, pH-sensitive liposomes were used to co-deliver paclitaxel and Bcl-2 siRNA in acidic conditions, with significant inhibition of tumor proliferation [54].

AuNPs were used for carrier-based chemotherapy in melanoma. Ultra-small gold nanoparticles conjugated to doxorubicin (Au-Dox) were investigated after intratumoural injection [55] and in cultured B16 melanoma cells [56], showing higher selectivity and enhanced effectiveness.

### 3.4. Gene Therapy

While cancer gene therapy represents a promising approach in the management of melanoma, one of the greatest challenges is represented by the development of high-performance delivery systems characterized by low toxicity and immunogenicity, as well as a high transfection efficiency. While viral carriers have also been approved for the treatment of melanoma, non-viral carriers using nanoparticles are under study.

#### 3.4.1. Intralesional Immunotherapy with Oncolytic Viruses

Talimogene laherparepvec (T-VEC) is an attenuated oncolytic herpes simplex virus that contains the granulocyte macrophage colony stimulating factor gene. In the randomized phase III trial OPTiM, which included patients with unresectable stage IIIB–IVM1c melanoma, Andtbacka R.H.I. et al. (2019) revealed the superiority of T-VEC compared to subcutaneous recombinant granulocyte-macrophage colony-stimulating factor (GM-CSF) regarding long-term efficacy and patient tolerance [57]. T-VEC was also associated with prolonged survival [57]. Interestingly, the vaccine seems to have a “bystander effect”, acting on injected lesions and also distant uninjected melanoma metastasis [58,59]. Complete responses were durable in time and were associated with prolonged survival [57].

#### 3.4.2. Nanoparticle Carriers for Gene Delivery

Sun Z. et al. (2018) tried to enhance the antigenic immune response by the co-encapsulation of GM-CSF with ovalbumin nanoparticles in a thermosensitive hydrogel [60]. They proved that the nanoparticle combination achieved superior immune effects [60].

Zhang Q. et al. (2019) studied the in vivo effects in a B16-F10 mouse melanoma tumor model of the tumor suppressor phospholysine phosphohistidine inorganic pyrophosphate phosphatase delivering its plasmid using a nanoparticle gene delivery system [61]. The nanocomplex inhibited melanoma growth with low toxicity [61].

Labala S. et al. (2016) investigated the efficiency of layer-by-layer chitosan-coated AuNPs as nano-carriers for the iontophoretic delivery of *STAT3-siRNA* for melanoma treatment [62]. The formulation provided higher stability and higher cellular uptake by tumor cells with the aid of clathrin-mediated endocytosis and, furthermore, inside the tumor cell, siRNA repressed the overexpression of STAT3 protein, favoring melanoma cell destruction [62]. The same research team later conjugated the AuNPs with imatinib, forming a co-delivery system, STAT3-siRNA–AuNPs and imatinib–AuNPs, which generated higher apoptosis in melanoma cells [63].

CNTs may also play a pivotal role in overcoming the biological barriers in siRNA delivery. Siu K.S. et al. (2014) developed a nanotube-based siRNA (small interfering RNA) topical delivery system [64]. SiRNA is an important axon decoder with a major impact on cancer growth and proliferation [64]. Its intracellular topical delivery is a challenge, mostly due to the hydrophilicity/lipophilicity balance and to the stability, surface charge, or size of the siRNA [64]. Single-walled CNTs, functionalized with succinated polyethylenimine (PEI-SA), were used for the topical delivery of Cy3-labeled siRNA into a melanoma mouse model [64]. Tumor progression was significantly reduced in 25 days [64].

### 3.5. Radiation Therapy

The role of radiation therapy in melanoma is mainly palliative, as it is recommended as the primary treatment for inoperable tumors and as adjuvant therapy in patients with desmoplastic melanoma [65]. Adjuvant radiation therapy has been shown to lower the risk of local regional recurrences [65,66]. Smaller doses can be used since randomized trials did not show relevant differences in control rates with larger fraction size compared with a smaller fraction size [65,67,68].

Radiotherapy alone has not been shown to improve patient overall survival [65]. However, radiation may increase antigen presentation, reduce immune escape mechanisms, and enhance the effect of immunotherapy [65,69]. Theurich S. et al. (2016) showed that the association of local radiation therapy or electrochemotherapy with ipilimumab led to an increase in overall survival [70].

Inadequate tumoral vascularization, hypoxia, and deficiencies of radiation absorption may limit the effect of radiotherapy [15]. Metal nanostructures, used as radiosensitizers, could improve the therapeutic action against melanoma. Several studies showed promising effects of AuNPs and PtNPs on X-ray absorption, as well as the efficacy against cancer cells [15,16].

Le Goas M. et al. (2019) improved internal radiotherapy with ^131^I by the conjugation of the radioisotope with polymer-grafted AuNPs [71]. The results were promising, with a significant increase in melanoma cell death in vitro and in vivo [71].

Daneshvar F. et al. (2019) combined X-ray radiotherapy with 808 nm diode laser photothermal therapy of melanoma B16/F10cells after their sensitization with PtNPs [15]. They observed an enhanced therapeutic action, with the efficient death of cancer cells [15].

### 3.6. Photothermal Therapy

Photothermal therapy (PTT) has recently emerged as a promising alternative for tumor targeting therapy. Nanoparticles have the ability to absorb long-wavelength light (usually near-infra-red light) and convert its electromagnetic energy into heat. After the bio-accumulation of nanoparticles into the tumor, the external irradiation with a laser light source will induce a destructive heating of the cancer cells [72].

Due to their capacity to effectively absorb near-infra-red (NIR) light and transform it into heat, AuNPs are extremely helpful in the photothermal therapy (PTT) of melanoma and other cancers [73]. Infrared light is used to make the electrons oscillate, then the energy from these oscillations spreads to the surrounding areas and the sudden temperature increase kills cancer cells [73].

A gold–ferrite nano-composite (GFNC) proved to be suitable in melanoma PTT ablation. After the injection of the gold nano-shell as a photothermal agent and upon laser irradiation in melanoma mice models, the tumor volume tumor size was substantially diminished when compared to the control groups [74].

In addition, CNTs are a promising approach in cancer hyperthermia. In a recent study, upon targeting melanoma cells in conjunction with photothermal therapy, the rate of cell death caused by CNT-mediated PTT ablation was explored on melanoma mice models [72]. After the intravenous injection of functionalized multiwalled CNTs (Polyethylene glycol-coated oxidized carbon nanotubes), the photothermal destruction of melanoma cells was highly enhanced, in contrast to the laser-mediated photothermal ablation alone [72].

Gorgizadeh M. et al. (2019) used carbon xerogel nanoparticles as a photoabsorber of 808 nm laser light in vitro and in vivo in a melanoma tumor-bearing mouse model with good biocompatibility and tumor control [75].

### 3.7. Photodynamic Therapy

Photodynamic therapy (PDT) is another alternative, two-step therapy that comprises the administration of a photosensitive drug to targeted cancer cells, followed by laser light application at a specific wavelength that activates the photosensitizer [76]. As a consequence, cytotoxic reactive oxygen species (ROS) and free radicals are formed, with the ability to damage blood vessels and consequently inhibit the tumor metabolism, to the extent of inducing a localized destruction of the targeted cancerous tissue [76]. The conjugation of nanoparticles to photosynthetic drugs improves the uptake of photosensitizers in targeted melanoma tissue and overall enhances PDT-mediated tumor destruction [76].

Gold nano-shells can also be used as photosensitizers in PDT. Due to their capacity to produce major reactive oxygen species for the photo-dynamic technique under NIR light, they consequently proved to suppress the proliferation of melanoma cells [77,78].

In a recent study by Mohammadi Z. et al. (2017), 5-aminolevulinic acid (5ALA), a photosensitizer limited by its low uptake by tumor cells, was conjugated with AuNPs and proved to be twice as efficient as 5ALA alone in promoting melanoma cell death [79].

## 4. Clinical Trials

Our literature research with the terms “nanoparticle” and “melanoma” led to the identification of 10 clinical trials, most of them on nanoparticle bound-paclitaxel. This compound is better tolerated because it eliminates the hypersensitivity reaction caused by the Cremaphor solvent [80]. The main adverse reaction to this medication is the peripheral neuropathy, with different grades of severity [80].

Hersh E.M. et al. (2010) aimed to determine the antitumor activity of the formulation ABI-007 (nanoparticle albumin-bound–paclitaxel) in patients with inoperable locally recurrent or metastatic melanoma and to determine the safety and tolerability of this drug [81]. The study (NCT00081042) was performed on two cohorts of 37 patients, each either previously treated with cytotoxic chemotherapy or chemotherapy-naïve [81]. Nab-Paclitaxel was well tolerated by both cohorts, with a median progression-free survival of 3.5 months versus 4.5 months and a median overall survival of 12.1 months versus 9.6 months [81].

Olencki T.E. et al. aimed to evaluate the response of patients with unresectable, metastatic uveal melanoma treated with paclitaxel albumin-stabilized nanoparticle formulation [82]. Only four patients were included in the study (NCT00738361) and the disease progressed in all at the time of the first scan [82]. Neither the median progression-free survival, nor the overall survival intervals were specified.

Kotschade L.A. et al. (2011) published the results of a phase II clinical trial (NCT00404235) that included patients diagnosed with unresectable stage IV melanoma who received a combined treatment with nanoparticle albumin-bound–paclitaxel and carboplatin [83]. The 76 patients were either chemotherapy naive or previously treated [83]. The median progression-free survival was 4.5 months versus 4.1 months, while the median overall survival was of 11.1 months versus 10.9 months [83]. Importantly, one complete response was achieved in the chemotherapy naive group [83].

Kottschade L.A. et al. (2013) studied the effectiveness of chemotherapy (temozolomide versus nanoparticle albumin-bound-paclitaxel and carboplatin) in combination with vascular endothelial growth factor (VEGF) inhibition (bevacizumab), in patients with unresectable stage IV melanoma [84]. The research group performed a randomized phase 2 study (NCT00626405) and included 93 chemotherapy-naive patients who were treated with the two regimes [84]. The results showed a slight superiority of the treatment strategy that included nanoparticle albumin-bound–paclitaxel with a median progression-free survival of 6.7 months versus 3.8 months and a median overall survival of 13.9 months versus 12.3 months [84].

In another clinical trial (NCT01300533) the highest safe dose of BIND-014 (docetaxel nanoparticles for injectable suspension) was investigated in 52 patients with advanced or metastatic cancer [85,86]. Only one patient with advanced melanoma was included in the trial and the medication was administered on days 1, 8, and 15 of a 28-day cycle [85,86]. Also, the researchers aimed to characterize the safety, pharmacokinetics, and antitumor activity of the medication [85,86]. One patient with cervical cancer showed a complete response [85,86]. The medication was well tolerated and had a pharmacokinetic profile distinct from docetaxel alone [85,86].

The results of a series of other completed clinical trials have not yet been published.

Ribas A. et al. conducted a phase I clinical trial (NCT00689065) that included 24 adult patients with solid tumors (including melanoma) refractory to standard care who were treated with targeted nanocomplex containing anti-R2 siRNA administered intravenously [87]. The results have not been published. Previously, the group analyzed tumor biopsies from melanoma patients obtained after treatment with the siRNA nanocomplex [88]. They showed that nanoparticles were present intracellularly in proportional amounts with the administered dose and that systemic siRNA can produce a specific gene inhibition [88]. The authors stated that the article represented the first report for systemically delivered nanoparticles of any kind [88].

Interesting results are expected to be published from the clinical trial coordinated by Markovic S.N. et al. (NCT02158520), which compared the response to the combined regimen of nab-paclitaxel and bevacizumab with immunotherapy (ipilimumab) [89]. The study included 24 patients with stage IV melanoma that could not be removed by surgery [89].

Stambuk H. et al. from the Memorial Sloan Kettering Cancer Center are currently investigating a new experimental dye-labeled particle (targeted silica nanoparticles of fluorescent cRGDY-PEG-Cy5.5-C dots) for sentinel lymph node mapping prior to surgery in 105 patients with head and neck melanoma, breast cancer, or colorectal cancer (NCT02106598) [90].

Svetomir M. et al. aim to include (NCT02020707) 36 patients diagnosed with stage IV melanoma that cannot be removed by surgery or with other types of gynecological cancers (cancer of the cervix, endometrium, ovary, fallopian tube, or peritoneal cavity) in a phase 1 clinical trial in order to determine the maximally tolerated dose of a regimen with nanoparticle albumin-bound–paclitaxel and bevacizumab [91].

## 5. Conclusions

Drug delivery platforms driven by nano-technology represent a standing example of precision medicine. As recent research has yielded promising results, we are confident that nano-structured materials will help empower the engineering of an end to melanoma mortality. By using nanotechnology, we are on the cusp of enabling personalized and targeted drug delivery in a way that could catalyze the next generation of therapeutics in melanoma.

The pre-exposure of melanoma tumors to nanotechnology or other compounds in order to trigger immunogenicity, followed by immunotherapy, might represent the focus of research in the coming years. Further studies, especially clinical trials, are needed to establish whether fighting melanoma on the nanoscale level represents the key of reaching a critical inflection point in mankind’s battle with metastatic melanoma.
